# Expression and Effects of High-Mobility Group Box 1 in Cervical Cancer

**DOI:** 10.3390/ijms15058699

**Published:** 2014-05-15

**Authors:** Xiaoao Pang, Yao Zhang, Heng Wei, Jing Zhang, Qingshuang Luo, Chenglin Huang, Shulan Zhang

**Affiliations:** Department of Obstetrics and Gynecology, Shengjing Hospital of China Medical University, Shenyang 110004, Liaoning, China; E-Mails: kellypang1015@hotmail.com (X.P.); zhangyao_1123@hotmail.com (Y.Z.); pp1015jay@163.com (H.W.); zhangjing-@hotmail.com (J.Z.); luoqingshuang1229@hotmail.com (Q.L.); huang-chenglin@hotmail.com (C.H.)

**Keywords:** high-mobility group box 1 protein (HMGB1), regulatory T cells (Tregs), cervical cancer, forkhead/winged helix transcription factor p3 (Foxp3), immunosuppression, serum squamous cell carcinoma antigen (SCC-Ag)

## Abstract

We investigated the significance of high- mobility group box1 (HMGB1) and T-cell-mediated immunity and prognostic value in cervical cancer. HMGB1, forkhead/winged helix transcription factor p3 (Foxp3), IL-2, and IL-10 protein expression was analyzed in 100 cervical tissue samples including cervical cancer, cervical intraepithelial neoplasia (CIN), and healthy control samples using immunohistochemistry. Serum squamous cell carcinoma antigen (SCC-Ag) was immunoradiometrically measured in 32 serum samples from 37 cases of squamous cervical cancer. HMGB1 and SCC-Ag were then correlated to clinicopathological characteristics. HMGB1 expression tends to increase as cervical cancer progresses and it was found to be significantly correlated to FIGO stage and lymph node metastasis. These findings suggest that HMGB1 may be a useful prognostic indicator of cervical carcinoma. In addition, there were significant positive relationships between HMGB1 and FOXP3 or IL-10 expression (both *p* < 0.05). In contrast, HMGB1 and IL-2 expression was negatively correlated (*p* < 0.05). HMGB1 expression may activate Tregs or facilitate Th2 polarization to promote immune evasion of cervical cancer. Elevated HMGB1 protein in cervical carcinoma samples was associated with a high recurrence of HPV infection in univariate analysis (*p* < 0.05). HMGB1 expression and levels of SCC-Ag were directly correlated in SCC (*p* < 0.05). Thus, HMGB1 may be a useful biomarker for patient prognosis and cervical cancer prediction and treatment.

## 1. Background

The human immune system has a close relationship with tumor occurrence and development. The incidence of tumors increases when immune function is inhibited. The down-regulation of human immunity and how this can lead to cervical cancer has become a research hotspot in recent years. High-mobility group box 1 protein (HMGB1) is critical for host immune suppression, and was found to be correlated protein in cervical squamous carcinoma [1]. Regulatory T cells (Tregs) have a wide range of immune suppression that inhibits anti-tumor responses, but the specific mechanisms by which they affect tumor behavior are not yet clear.

HMGB1, a nonhistone, DNA-binding protein, is needed for regulating gene transcription. It is actively released via cytokine stimulation and passively secreted upon cell death [2]. HMGB1 is an endogenous signal referred to as a damage-associated molecular pattern (DAMP) molecule that is released from tumor cells and thought to be related to various inflammatory disorders [3]. HMGB1 has many biological functions. It acts as an extracellular signaling molecule during cell migration, inflammation, cell differentiation, and tumor metastasis, and it mediates infection, injury and inflammation responses, promoting cell autophagy, inducing cell death, and activating innate immunity as signal conditioning factors [4]. Overexpression of HMGB1 was discussed in various types of malignancy, containing colorectal carcinoma, breast cancer, hepatocellular carcinoma, pancreatic cancer and prostate carcinoma [5–9]. Overexpression of HMGB1 has also been observed in cervical cancer and a correlation has been found between HMGB1 expression and malignant tumor phenotypes, such as metastasis and recurrence [3]. In addition, high levels of HMGB1 expression have been found to be a significant predictor of early prognosis in recurrent cervical SCC patients as well as an independent prognostic factor for recurrent cervical cancer outcomes [10]. In summary, HMGB1 has a crucial role in the processes involved in chronic inflammation caused by HPV infection in cervical cancer and may contribute to tumor progression. However, the specific mechanisms underlying this are still unclear and merit further study.

Accumulating amounts of evidence have demonstrated that regulatory T cells (Tregs), which express the transcription factor forkhead box P3 (FOXP3) are usually suppressive in the immune system. Some reports have demonstrated that many disease models were involved in several subtypes of Tregs [11]. Studies have shown that Tregs have a wide range of immunosuppressive effects both *in vitro* and *in vivo* and that it inhibits the activation and proliferation of CD4 + Th1 cells and other immune cells through direct surface contact with membrane molecules and through secretion of interleukin-10 (IL-10) and transforming growth factor-β (TGFβ) [3]. The relative number of Langer’s cells was shown to decrease significantly in local cervical tissue as the level of Foxp3 protein expression increased. This preliminarily confirmed that Tregs are associated with local cervical immune regulation [12]. It also indicates that the expression of Tregs is positively correlated with the pathological processes associated with cervical cancer, helping cervical cancer cells to evade the antitumor immune response, and promoting cervical cancer formation.

One study conducted in a tumor-burdened rat model of breast cancer indicated that tumor cell-derived HMGB1 can suppress a naturally acquired immune effector cell (CD8) or cytokine-dependent (IFN-γ-dependent) antitumor response, probably by enhancing tumor-associated Tregs to produce IL-10, which is necessary for immune suppression *in vitro* and *in vivo* [13]. In one study of a tumor-burdened rat model after thermal injury, excessive release of HMGB1 was found to stimulate splenic Tregs [14]. One possible mechanism underlying this could be binding to the receptor on the surface of Tregs, called RAGE, suppressing the T lymphocytes immune function in the form of a shift from Th1 to Th2 in burn injury models. Tregs activated by HMGB1 downregulated nuclear factor-kappa B signaling in T lymphocytes, which inhibited the function of T lymphocytes and polarized Th1 cells to Th2 cells [14]. Some studies indicate that HMGB1 may modulate immunity conferred by T cells by influencing the proliferation of effector T cells, as well as contributions to cell polarization and IL-2 secretion [15]. However, whether Treg cells can be activated by HMGB1 in cervical cancer is unclear, and how HMGB1 can influence effector T cells needs further research. This merits speculation that through binding to the RAGE receptor, HMGB1 may help cervical cells evade immunosuppression, and accelerate the process of “HPV infection to CIN to cervical cancer” by influencing T cell-mediated immunity.

SCC antigen (SCC-Ag) is a squamous carcinoma biomarker. Some studies have shown that high levels of expression of serum SCC-Ag may indeed be associated with advanced disease stages, involvement of lymph nodes, tumor size, and poor response to treatment [16]. Identifying the serum level of SCC-Ag is necessary for early diagnosis and induction of clinical treatment.

Therefore we sought to identify whether HMGB1 is overexpressed in cancer tissues compared with normal epithelia tissues. Also, we sought to investigate any connection between the expression of HMGB1 and FOXP3, IL-2, IL-10, as well as serum levels of SCC-Ag. What is more, all relationships with clinicopathological parameters were also analyzed.

## 2. Results

### 2.1. Patient Characteristics

The 100 patients examined here, both controls and those with pathological cervical neoplasms, included 51 with CIN, 37 with ICC, and 12 with normal squamous epithelium. Patient characteristics are listed in Table 1.

### 2.2. Immunohistochemistry

**Figure 1.**
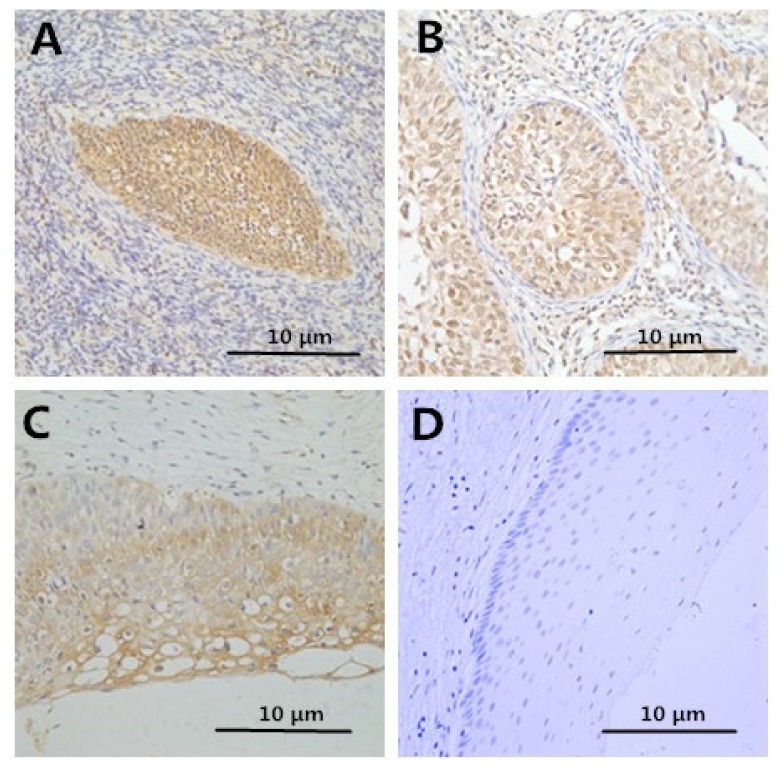
Immunohistochemical staining of HMGB1 in cervical samples. HMGB1 staining in cervical samples (**A**–**D**). The analysis showed HMGB1 expression in tumor cells was positively stained for nuclei (**A**); clear staining in CIN III (**B**); and potent staining in CIN I–II (**C**); weak staining in normal (**D**). Image magnifications are 400×.

Positive HMGB1 immunoreactivity was detected in 36/37 (97.3%) cancer cases, 24/25 (96%) CIN3 cases, 9/26 (34.6%) CIN1-2 cases and 0/12 (0%) control cases. HMGB1 staining in a representative case of cervical cancer is shown in Figure 1; HMGB1 was chiefly observed in carcinoma cell cytoplasm and nuclei. HMGB1 had a strong staining in carcinoma tissues compared to normal tissues (*p* < 0.05). These results indicate that HMGB1 is overexpressed in cervical carcinoma cells and may increase as cervical cancer progresses.

**Figure 2.**
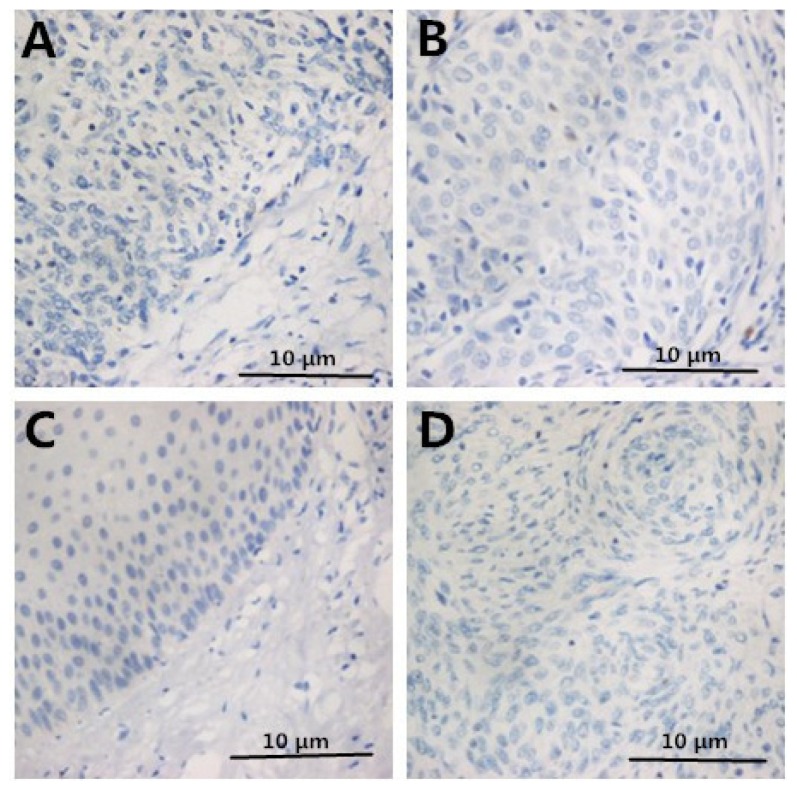
Negative control staining of HMGB1, FOXP3, IL-2, and IL-10 is displayed in sub-figures **A**–**D**.

The protein expression levels of Foxp3 and IL-10 increased along with the progression of pathology of cervical disease. However, the protein expression level of IL-2 decreased (Table 2 and Figure 1). The negative control staining of HMGB1, FOXP3, IL-2, and IL-10 is displayed in Figure 2. Thus, HMGB1 may contribute to the cervical cancer progression via enhancing Tregs.

### 2.3. Correlation between Clinicopathologic Factors and HMGB1 Expression

The grade of HMGB1 expression was found to be significantly closely associated with histopathology FIGO stage, and lymph node metastasis. HMGB1 expression increased from normal to CIN to ICC specimens, suggesting HMGB1 expression grading is directly correlated with the malignant potential of cervical neoplasia. However, no significance was found between the HMGB1 expression and cell differentiation or histopathology. In contrast, HMGB1 expression grading was related with lymph node metastasis and FIGO stage (Table 3). These findings suggest that HMGB1 may be a useful prognostic and diagnostic indicator of cervical carcinoma.

### 2.4. Expression of FOXP3, IL-2, IL-10 in Specimens

FOXP3 in ICC and CIN were significantly enhanced compared to normal control. We also observed that IL-10 in ICC was upregulated compared to CIN or normal tissues.

**Figure 3.**
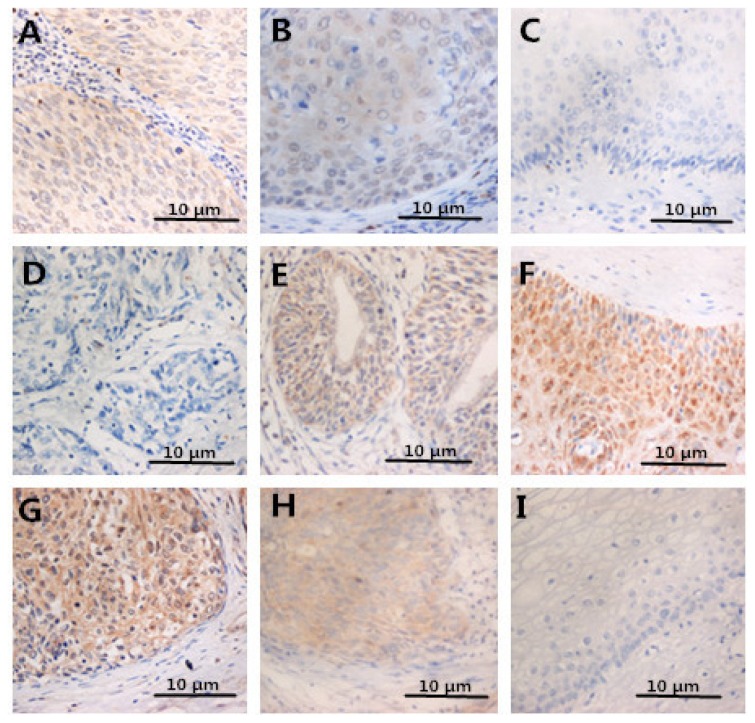
Immunohistochemical staining of FOXP3 in cervical samples (**A**–**C**); staining of IL-2 in cervical samples (**D**–**F**); staining of IL-10 in cervical tissues (**G**–**I**).

**Figure 4.**
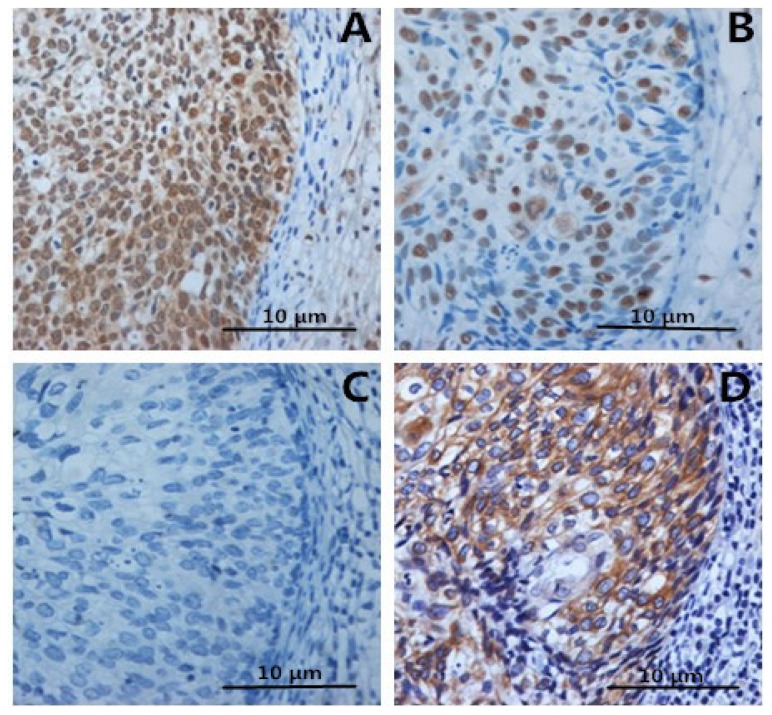
As shown in immunostaining of **A** (HMGB1), **B** (Foxp3) and **D** (IL-10), most of Foxp3+ Tregs are IL-10-positive. Tregs have been known to be IL-2-negative, as shown in **C**.

However, no significant differences were noted between CIN1-2 and normal epithelium. In contrast, IL-2 in ICC and CIN was lower than in controls (Figures 3 and 4). After analysis, we found that correlations between HMGB1 and FOXP3 or IL-10 expression were significantly positive (both *p* < 0.05). In contrast, there was a negative correlation between HMGB1 and IL-2 expression (*p* < 0.05). These results suggest that HMGB1 expression might activate Tregs or facilitate Th2 polarization to promote immune evasion of cervical cancer cells.

### 2.5. The Prognostic Significance of HMGB1 Protein in Cervical Carcinoma Tissue Samples

**Figure 5.**
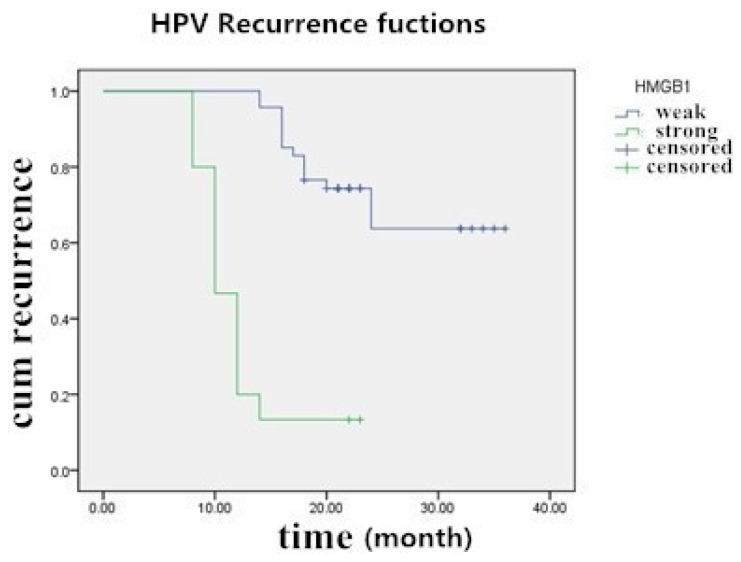
Kaplan-Meier survival curve of 62 patients with cervical cancer and CIN III according to HMGB1 protein staining.

In the course of the assessment period from 23 December 2010 to 23 January 2014, among the 62 cervical carcinoma and CIN3 patients who had been HPV positive before the surgery, 26 (41.94%) experienced recurrent HPV infection, 6 (9.68%) were lost during follow-up, and 30 (48.39%) did not acquire the HPV infection again. For patients with strong expression of HMGB1, the median follow-up time for the entire group of 62 patients was 19.4 months (range of 8–36 months). The estimated recurrence rate and the mean ± SD of the median recurrence time were 81.25% and 12.13 ± 1.18 months, respectively, and for patients with weak or negative HMGB1 expression, they were 28.26% and 29.75 ± 1.64 months respectively. In this way, patients strongly expressing HMGB1 had significantly greater rates of recurrence of HPV infection than patients with weak expression (*p* < 0.05, log-rank test) (Figure 5).

### 2.6. The Relationship between SCC-Ag and HMGB1 Expression

Serum SCC-Ag in patients without pretreatment ranged from 0.6–35.5 ng/mL (mean, 4.25 ng/mL). We set classification value at 1.5 ng/mL, 18 patients (56.25%) had 1.5 ng/mL or lower serum SCC-Ag, and 14 patients (43.75%) had greater than 1.5 ng/mL. In SCC groups, SCC-Ag and HMGB1 expression had a positive correlation. (*r* = 0.517, *p* < 0.05; Table 4).

## 3. Discussion

As is widely reported around the world, the expression of HMGB1 protein level was higher in almost all tumors (especially in epithelial tumors) than in healthy tissues. HMGB1 is a cytokine and also a growth factor. Necrosis of tumor cells can release HMGB1, which can cause chronic inflammation in the tumor microenvironment and help tumor cells survive, grow and metastasize [17]. Primarily two signaling pathways mediate the proinflammatory effects of HMGB1. Importantly, the Ras/MAPK pathway, which is involved in MAPKs phosphorylation and activates NF-kB, induceing inflammation and immune cell migration. NF-κB affects the cell cycle directly leading to the occurrence of cancer [18]. Previous studies have shown that NF-kB may be involved in promoting tumor metastasis by regulating cytokines such as TNF, IL, MMP-9, and ICAM [19,20]. HMGB1 could bind to RAGE, which belongs to the immunoglobulin superfamily, a kind of transmembrane protein, playing an important role in diseases such as diabetes and inflammatory diseases. Wang [21] has found that HMGB1 may be binding to RAGE on the surface of dendritic cell (DC) cells to regulate TNF-a, IL-2, and IL-6 expression. The MAPK pathway may be one of the major intracellular signal transduction pathways inducing RAGE regulation of gene expression [22]. The MAPK pathway may also participate in the process of maturation of Tregs induced by inflammation [23]. However, HMGB1 expression and function may not be the same in certain tumor cells and healthy cells. In the process of tumor progression, HMGB1 has been shown to inhibit dendritic cells in the tumor microenvironment, indicating that HMGB1 may participate in tumor evasion and in the promotion of cancer development [24]. In one study of a tumor-burdened rat model after thermal injury, the release of excessive HMGB1 after burn injury was found to activate splenic Treg cells. The most likely mechanism was binding to the Treg surface receptor, RAGE, thus suppressing T lymphocytes in favor of shifting from Th1 cells to Th2 cells [14]. Some studies has found that HMGB1, Tregs, and CD88 on the surface of DC cells were significantly positively correlated in spleen tissues, which indicated that HMGB1 might participate in the process of maturation of spleen DC cells through mediating Tregs after serious burn [14]. Studies from Dumitriu *et al*. [25] and Rovere-Querini *et al.* [26] has found that the conditioned medium which contains HMGB1 may promote the maturation of dendritic cells and promote the proliferation of T lymphocytes. Recent studies have showed that various related TOLL-like receptor (TLRs) could be expressed on the surface of Tregs [27], and related studies also showed that HMGB1 might bind to RAGE, TLRs to conduct the activation of cells [27]. Cytokines such as IL-2, IL-6, and IL-15 also participate in mediating the proliferation and immunosuppression of Tregs [27]. Thus we assume HMGB1 may be binding to the TLRs on Treg surfaces to regulate TLR expression and to activate related signal pathways and then activate NF-κB in the nucleus, leading to the release of different cytokines such as IL-4, IL-10, and IL-2. One study conducted in a tumor-burdened rat model of breast cancer indicated that interactions between Tregs cells and cytokines released from Tregs in this process resulted in T cells shifting from Th1 cells to Th2 cells [14]. There is also evidence to suggest that HMGB1 could not stimulate nature killer cells (NK cells) induced by Tregs releasing INF-γ when coordinated with IL-2, IL-1, and IL-10 [28]. Thus we speculate that the inhibition of T lymphocytes after burn injury was not caused directly by HMGB1 stimulation, but, may have been mediated by Tregs. The cytokine microenvironment was crucial in deciding the differentiation of T lymphocytes, and the upregulation of IL-4, and IL-10 played a key role in this process. Whether this kind of shift takes place in cervical cancer is currently unclear. To access the correlation between the expression of HMGB1 and immune regulation, the level of HMGB1 and the expression of Treg-related FOXP3, IL-2, and IL-10 protein were examined in cervical tissues. The present study demonstrated that HMGB1 might be correlated positively with Foxp3 expression and both of them were significantly enhanced in cervical cancer tissues. The lesions were found to be more likely to progress in cervical tissues with positive expressions of both HMGB1 and FOXP3. In contrast, in cervical tissues that were negative for both types of expression, the lesions underwent spontaneous regression. In this way, HMGB1 may suppress the human immune function by up-regulating Tregs, which facilitates infection and the growth of cervical-cancer cells. Enhanced Treg activity has been shown to regulate T lymphocytes cell-mediated immunity in the tumor microenvironment [14]. The IL-2 cytokine is mainly produced by Thl cells during immune responses, and IL-10 is mainly produced by Th2 cells and Tregs as an anti-inflammatory cytokine. The data shown here suggest that HMGB1 may directly activate Tregs by binding to RAGE, which promotes IL-10 production in cervical cancer or helps to shift Th1 cells to Th2 cells and assist in tumor evasion and development. However, the specific and exact mechanism by which HMGB1 stimulates Tregs and its role in shifting of Th1 cells to Th2 cells in cervical cancer remains obscure. These questions will be addressed in our next research project.

The correlations between HMGB1 expression and clinicopathologic factors were then assessed to determine whether HMGB1 expression could be used as a risk predictor of malignancy and metastasis. These data indicate that HMGB1 expression increased with the advancement of FIGO stage and lymph node cervical cancer metastasis. These data indicate that HMGB1 may be critical for cervical carcinoma progression. However, HMGB1 protein overexpression was not found to be correlated with age, differentiation or histology of cervical cancer in patients. A larger number of cervical cancer samples need to be analyzed to substantiate these results.

Generally, SCC-Ag is a prognostic and predictive maker of cervical cancer. Previous studies were expanded here by evaluating the correlation between HMGB1 expression and serum SCC-Ag [29,30]. SCC-Ag was shown to have a positive relationship with HMGB1 expression, indicating that HMGB1 may acte as a biomarker for the evaluation of cervical cancer prognoses and biological behaviors.

## 4. Materials and Methods

### 4.1. Specimens

One hundred paraffin uterine cervical samples were obtained from operations performed from 2010 to 2012 in the department of Gynecology and Obstetrics of Sheng Jing Hospital Affiliated to China Medical University. Among the 100 HPV-positive cases studied, there were 51 cases of CIN, 37 cases of invasive cervical cancer (ICC) and 12 normal squamous epithelial tissues. Normal tissues were obtained from patients with benign uterine tumors who underwent hysterectomy. The median patient age was 44 years (range: 19–68 years). The Ethics Committee of the Shengjing Hospital Affiliated to the China Medical University approved the study. After treatment, the 37 cervical carcinoma patients were followed up from 23 December 2010 to 23 January 2014. All patients underwent follow-up visits each month for the first year, every third month in the second year, and twice a year thereafter. Among these 37 patients, serum samples were collected from 32 patients with cervical squamous cell carcinoma to measure serum SCC-Ag prior to treatment.

Histopathological diagnoses were based on the WHO classification and clinical stages were in accordance with the International Federation of Gynecology and Obstetrics (FIGO) criteria. All the tumors were primary and the patient information and follow-up data were complete. No chemical treatment was used in any patient before surgery.

### 4.2. Immunohistochemistry

The tissues were embedded in paraffin and fixed in 4% formaldehyde, and serial sections were used. We used mouse anti-HMGB1 at 1:40 (R&D SYSTEMS Inc., Minneapolis, MN, USA); mouse anti-Foxp3 at 1:100; rabbit anti-IL-2 at 1:320; rabbit anti-IL-10 at 1:100. Phosphate-buffered saline (PBS) was substituted for the primary antibody in the negative control. Serial sections were used for all single staining to show that IL-2, IL-10, and Foxp3 signals were also positive for CD4 T cells. After overnight incubation at 4 °C and 3 washes in PBS were performed. The procedure was based on the SP kit system (Zhongshan-Golden Bridge Biotechnology, Ltd., Beijing, China).

### 4.3. Grading of Immunostaining

Two researchers who were blinded to the patients’ materials examined the immunostained slides with microscopy in a bright-field.

To assess immunostaining data for HMGB1, FOXP3, IL-2, and IL-10, an immunostaining scoring system corresponding to total staining intensity was as follows: (strong staining scores 3; moderate staining scores 2; weak staining scores 1; no staining scores 0). Scores for the relative numbers of positive cells were as follows: (>75% of cells were positive = 4+; 51%–75% of cells were positive = 3; 26%–50% of cells were positive = 2; less than 25% of cells were stained positive = 1; no positive cells = 0).

### 4.4. Immunoradiometric Assay

SCC kit (Imx, Abbott Diagnostics, Lake Forest, IL, USA) was used to detect the serum levels of SCC-Ag. We set the basic classification at 1.5 ng/mL. The sensitivity of the detection was about 0.1 ng/mL.

### 4.5. Statistical Analyses

We analyzed the comparison of protein expression and SCC-Ag levels among different pathological lesions using χ^2^ test. A Spearman rank correlation test assessed correlations of HMGB1 with FOXP3, IL-2, IL-10 protein expression and serum SCC-Ag. We also used the Kaplan-Meier method to conduct the univariate overall survival analysis. Survival rate differences were performed with the log-rank test. We analyzed all the statistics using SPSS 17.0 software (SPSS, Inc., Chicago, IL, USA, 2009). We used 2-sided tests and set 0.05 as cutoff value.

## 5. Conclusions

Data collected here demonstrated that the expression of HMGB1 in cervical lesions increased with tumor progression. It is speculated here that HMGB1 might help tumor cells evade immune surveillance and could be a useful negative prognostic indicator of cervical carcinoma. Further studies investigating the mechanisms underlying the action of HMGB1 in cervical carcinoma may be warranted.

## Figures and Tables

**Table 1.** Clinical and pathological characteristics.

Characteristics	Number of cases (%)
Range	19~68 (years)
Average	44 (years)
Samples	
Normal squamous epithelial specimens	12 (12)
Cervical intraepithelial neoplasms (CIN)	51 (51)
CIN I/II	26 (51)
CIN III	25 (49)
Invasive CC (ICC)	37 (37)
Well-differentiated (WICC)	7 (18.9)
Moderately differentiated (MICC)	24 (64.9)
Poorly differentiated (PICC)	6 (16.2)
Histology	
Squamous cell carcinoma (SCC)	31 (83.8)
Adenosquamous cell carcinoma (ACC)	6 (16.2)
FIGO stage	
Ia	3 (8.2)
Ib	18 (48.6)
IIa	15 (40.5)
IIb	1 (2.7)
Lymph nodes metastasis (LN)	
Absent	26 (70.3)
Present	11 (29.7)

**Table 2.** Relationship between HMGB1 expression and clinicopathological factors.

Characteristics	*n*	HMGB1				*p*

		−	+	++	+++	
Normal	12	10	2	0	0	<0.05
CIN	51	2	21	28	0	
CIN I/II	26	1	16	9	0	<0.01
CIN III	25	1	5	19	0	<0.05
ICC	37	0	1	20	16	
WICC	7	0	1	6	0	
MICC	24	0	0	10	4	0.717
PICC	6	0	0	4	2	
Histology						
SCC	31	0	0	17	14	0.353
ACC	6	0	1	3	2	
FIGO stage						
I	21	0	1	19	1	<0.05
II	16	0	0	1	15	
LN metastasis						
Absent	26	0	1	17	8	0.02
Present	11	0	0	3	8	

**Table 3.** Association between HMGB1 and FOXP3, IL-2, IL-10 expression in cervical samles.

Pathological grade	*n*	HMGB1				FOXP3			
	
		−	+	++	+++	−	+	++	+++
Normal	12	10	2	0	0	10	2	0	0
CINI/II	26	1	16	9	0	2	18	6	0
CINIII	25	1	5	19	0	1	5	17	2
ICC	37	0	1	20	16	0	3	19	15

*r* = 0.829, *p* < 0.05									

Pathological grade	*n*	IL-2				IL-10			
	
		−	+	++	+++	−	+	++	+++

Normal	12	0	0	2	10	10	2	0	0
CINI/II	26	0	2	18	6	3	10	12	1
CINIII	25	2	19	2	2	0	5	17	3
ICC	37	28	6	2	1	0	4	16	17
			
		*r* = −0.587, *p* < 0.05				*r* = 0.746, *p* < 0.05			

**Table 4.** Relationship between SCC-Ag and HMGB1 expression.

HMGB1	*n*	SCC-Ag	

		<1.5 ng/mL	>1.5 ng/mL
+/++	18	13 (72.2)	5 (27.8)
+++	14	5 (35.7)	9 (64.3)
Total	32	18	14

		*r* = 0.517, *p* < 0.01	
